# Camera-based photoplethysmography in an intraoperative setting

**DOI:** 10.1186/s12938-018-0467-7

**Published:** 2018-03-14

**Authors:** Alexander Trumpp, Johannes Lohr, Daniel Wedekind, Martin Schmidt, Matthias Burghardt, Axel R. Heller, Hagen Malberg, Sebastian Zaunseder

**Affiliations:** 1 Institute of Biomedical Engineering, TU Dresden, Fetscherstraße 29, 01307 Dresden, Germany; 2 Department of Anesthesiology and Intensive Care Medicine, University Hospital, TU Dresden, Fetscherstraße 74, 01307 Dresden, Germany

**Keywords:** Camera-based photoplethysmography, Intraoperative monitoring, Remote monitoring, Level set methods, Spatial homogeneity

## Abstract

**Background:**

Camera-based photoplethysmography (cbPPG) is a measurement technique which enables remote vital sign monitoring by using cameras. To obtain valid plethysmograms, proper regions of interest (ROIs) have to be selected in the video data. Most automated selection methods rely on specific spatial or temporal features limiting a broader application. In this work, we present a new method which overcomes those drawbacks and, therefore, allows cbPPG to be applied in an intraoperative environment.

**Methods:**

We recorded 41 patients during surgery using an RGB and a near-infrared (NIR) camera. A Bayesian skin classifier was employed to detect suitable regions, and a level set segmentation approach to define and track ROIs based on spatial homogeneity.

**Results:**

The results show stable and homogeneously illuminated ROIs. We further evaluated their quality with regards to extracted cbPPG signals. The green channel provided the best results where heart rates could be correctly estimated in 95.6% of cases. The NIR channel yielded the highest contribution in compensating false estimations.

**Conclusions:**

The proposed method proved that cbPPG is applicable in intraoperative environments. It can be easily transferred to other settings regardless of which body site is considered.

**Electronic supplementary material:**

The online version of this article (10.1186/s12938-018-0467-7) contains supplementary material, which is available to authorized users.

## Background

In the last decade, a novel optical measuring technique called camera-based photoplethysmography (cbPPG) has gained a lot of attention. The technique permits the remote extraction of cardio-respiratory signals using conventional video cameras [1, 2]. Similar to the classical photoplethysmography (PPG), the signals are mainly modulated by blood volume changes in the cutaneous microvasculature [3]. However, cbPPG has the benefit of allowing a spatial assessment of the microcirculatory perfusion which provides a new diagnostic value [4].

For a broad and convenient application of cbPPG, a region of interest (ROI) has to be detected and tracked automatically at suitable skin regions in the video recordings. The efficiency of ROI selection eventually determines the quality and validity of the extracted plethysmograms and is, therefore, a crucial step. Facial regions are a good candidate since they are most often accessible and because the cutaneous perfusion is relatively high there [5]. In the past, the vast majority of works used face or facial landmark detection combined with subsequent redetection or tracking of selected features to (pre-)define ROIs in the context of cbPPG (e.g. [6–10]). However, such approaches rely on the visibility of certain anatomical areas and might fail if the face is partly occluded or rotated. Even if they succeed, a selected ROI could still be blocked, for example by hair. These problems may not be relevant in controlled environments, like the laboratory, but have to be considered in clinical or public settings [11, 12].

One way to reduce the dependence on facial features is to include the time component in the selection process (e.g. [8, 13–16]). For that purpose, the image or a predefined ROI is blurred or divided into small sub-ROIs. The extracted signals from those pixels/sub-ROIs are then assessed for further use in terms of variations related to the cardiac cycle. A lot of those approaches nevertheless involve an initial ROI definition. Furthermore, they all rely on a distinct manifestation of the cardiac pulsation, which is most likely dominant for young and healthy subjects, but certainly diminished in older and vascular diseased subjects, and consequently, hard to determine when using small image regions [2, 3]. Another way to select facial ROIs is to utilize skin classifiers which detect proper areas based on the skin’s appearance in various color spaces. Most of those works, however, still combine the classifiers with face or facial landmark detection (e.g. [17–19]). There are only a few exceptions that either not exploit the found skin regions or focus, again, on the time component (signal processing) to obtain valid cbPPG signals and vital parameters [20–22].

Recently, Moço et al. [23, 24] revealed how ballistocardiographic (BCG) effects degrade the wanted blood volume signal in cbPPG. The group showed that for the face, these effects are mainly present if the light source is not orthogonally directed towards the skin surface and the ROI is not homogeneously illuminated. For this reason, the selection of spatially homogeneous ROIs is essential to achieve pure cbPPG signals. Previous approaches, which in some way considered the ROI’s homogeneity, employed intensity thresholds, exploited regional means and standard deviations or clustered areas based on the lightness component [9, 25, 26]. For the eventual application, all those methods depend on an initial face detection.

In this paper, we propose a novel and fully automated ROI selection method that utilizes level set segmentation to minimize the influence of BCG artifacts. The method (i) does not rely on the detection of anatomical features, (ii) chooses and tracks visible skin regions which are homogeneously illuminated, and (iii) solely operates on the image plane without being reliant on the presence of temporal variations related to the cardiac cycle. We demonstrate the applicability of our method for the face area of 41 patients which were recorded during surgery using a multi-camera setup. The performance was evaluated with respect to the quality of extracted cbPPG signals and correctly detected heart rates (HRs). To the best of our knowledge, only Rubīns et al. [27, 28] applied cbPPG so far in an intraoperative environment analyzing the inner hand area.

## Methods

### Data and setup

Our study was conducted at the Department of Anesthesiology and Intensive Care Medicine (University Hospital Carl Gustav Carus) in Dresden. It was authorized by the Institutional Review Board at TU Dresden (IRB00001473, EK168052013) and was in accordance with the Helsinki Declaration. We included 41 elderly patients in the cbPPG analyses of whom each had to give written consent. All clinically relevant information about the volunteers such as their medical history was logged. We recorded the patients for approximately 30 min while they underwent surgery on the torso or extremities. Important events during the surgical procedure and interventions by anesthetists were also tracked. Table 1 summarizes the most important characteristics of the patient group. As depicted, almost half of the participants had a relevant degree of vascular disease (e.g. stenosis, varicosis, thrombosis, hypovolemia, artery occlusive disease). Consequently, the strength of the blood volume pulse in the microvasculature might have been affected limiting the extraction of valid cbPPG signals.

**Table 1:** Important characteristics of the patient group

Characteristic	Value
Age (in years)	65.2 ± 12.0
Female/ male (number)	17/24
Body mass index (in kg/m^2^)	26.1 ± 4.6
NYHA (number)^a^	
0—not examined	4
1—no problems	35
2—irrelevant problems	0
3—relevant problems	2
Vascular system (number)^a^	
0—not examined	0
1—no problems	20
2—irrelevant problems	2
3—relevant problems	19
Duration surgery (in min)	157.3 ± 99.9
Duration video recording (in min)	32.0 ± 7.2

^a^The categories stem from the ANDOK$^{\mathrm {live}}$ protocol. For the NYHA (New York Heart Association) classification, they describe the relevance of assistance based on the degree of heart failure

**Fig. 1:**
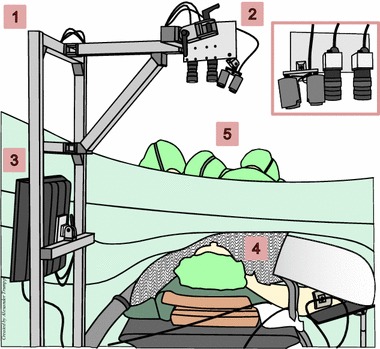
CbPPG setup during surgery. (1) Construction with adjustable arm for the sensing system. (2) Sensing system (enlarged on the right) including NIR illumination, NIR camera, and RGB camera. (3) Recording PC. (4) Patient (face directed towards the cameras). (5) Surgeons and clinical staff

For video recording, we used a mobile measuring system that was already applied successfully in another clinical study [11, 12]. The system consists of a medical PC (ACL OR-PC 19) and a sensing component which are both mounted on a movable constructional framework (see Fig. 1). The sensing component encompasses two cameras (IDS Imaging Development Systems GmbH), a monochrome camera (UI-3370CP-NIR-GL) and an RGB camera (UI-3370CP-C-HQ), and a near-infrared (NIR) light source with four LED spots (Kingbright BL106-15-29). In combination with an additional NIR bandpass filter (MidOpt BP850) at the monochrome camera, the light source permitted a controlled measurement in the non-visible range (880 nm). We equipped both cameras with lenses by Schneider-Kreuznach (Cinegon 16/1.8) and set them up to a color depth of 12 Bit, a frame rate of 100 fps, and a resolution of $320\times 420$ pixels. Before each recording, the sensing component was aligned at a distance between 0.5 and 1 m over the head of the patient who was in a supine position (see Fig. 1). Due to general anesthesia, the subject was unconscious during the measurement. The illumination for the RGB video was defined by the surgical light above the table and by the room’s fluorescent lamps. For reference purposes, we also synchronously stored physiological signals from the patient monitor (e.g. photoplethysmogram) on our medical PC.

For our analyses, we aimed at using facial areas as ROI. However, the following obstacles in the intraoperative setting challenged the ROI selection process:

- Face was often partly occluded by surgical drape
- Patient was moved by clinical staff
- Measuring stand was relocated
- Staff reached into recording area
- Operation table was readjusted in height
- Illumination varied due to moving staff
- Patient moved due to surgical procedure.

In the next section, we describe the developed method that is able to tackle those problems.

### Image processing

The ROI selection algorithm, which is presented here, is an enhanced and more complex version of an approach that we successfully applied to recordings (only single camera) of patients in an intensive care unit [12]. The new algorithm allows to process the RGB and NIR video stream simultaneously. For that purpose, the two streams were synchronized leading to a frame-wise assignment in which the time component of both streams can be expressed by the same frame number ${k}$. An image pair to a certain instant ${k}$ is then representable by the four channels $I_R(\mathbf {x}),$ $I_G(\mathbf {x}),$ $I_B(\mathbf {x}),$ $I_{N}(\mathbf {x})$ (red, green, blue, NIR) with $\mathbf {x}=(x,y)$ being the spatial component.

#### Skin cassification

In our setup, common face detection algorithms, as used in [6, 7, 9], eventually failed due to the limited visibility of required features. To detect suitable regions that potentially provide physiological information, we employed a skin classifier by Jones and Rehg [29] on the (first) RGB image. The classifier has to be built once and is then generally applicable. First, two RGB histograms, one for the class $skin$ and one for $\lnot skin$ were constructed using over 13,000 labeled skin and non-skin color pictures that were made available by the authors. Second, the conditional probability density functions $p({\mathbf c} | skin)$ and $p({\mathbf c} |\lnot skin)$ were calculated by normalizing the histograms on the total number of counts. Eventually, the classifier could be derived from the Bayesian decision rule [30]. A pixel was classified as skin if [29]

1 $$\begin{aligned} \frac{p({\mathbf c} | skin)}{p({\mathbf c} |\lnot skin)} \ge \theta \end{aligned}$$

where ${\mathbf c}$ is the pixel’s RGB value and $\theta$ a threshold which determines the ratio between the true positive and false positive classification rate. We found $\theta = 5$ to be a good trade-off. Before skin detection, we adjusted the image intensity^1^ because we discovered this step to boost the classifier’s performance.

#### Segmentation

**Fig. 2:**
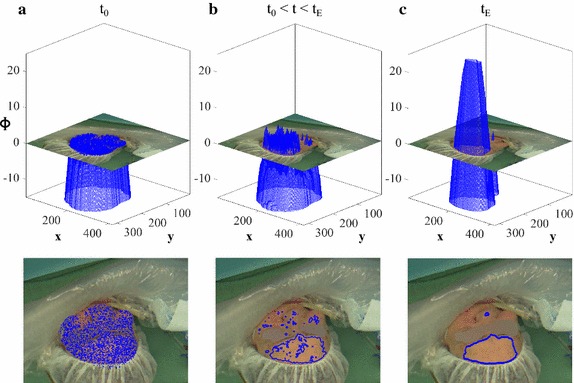
Example for a segmentation process using level set methods.** a** Initialization point.** b** Point during segmentation.** c** Point when process has converged. The inside region $\Omega _1$ and the outside region $\Omega _2$ are implicitly described and changed by $\Phi.$ The contour $\Phi =0$ is depicted separately in the images below the graphs. Please note that *t* represents the segmentation time for an image and does not refer to the time component in the videos. The eye section was blurred if it was visible

Since the classifier operates on a pixel level and does not take any local distributions into account, the outcome is usually insufficient and may not leave homogeneously illuminated skin regions (see Fig. 2a). To deal with this problem, we applied a segmentation approach by Brox et al. [31] which utilizes level set methods.

##### Level set methods for segmentation

Level set methods allow to describe an evolving segmentation contour *C* in an implicit manner using a function $\Phi ({\mathbf x},t)$ [32]. For a two-phase segmentation, there is an inside region $\Omega _1$ and an outside region $\Omega _2.$ Let $\Omega _1$ be an optimal ROI and $\Omega _2$ non-suitable skin areas and the background (whole image region $\Omega = \Omega _1 \cup \Omega _2$). As $\Omega _1$ might consist of numerous subregions that are not connected, an explicit description is challenging. This task is much easier when $\Phi$ is employed to implicitly describe the image plane (see Fig. 2): $\Phi >0 \Rightarrow \Omega _1,$ $\Phi <0 \Rightarrow \Omega _2,$ $\Phi =0 \Rightarrow C$ (’$\Rightarrow$’ denotes ’implies’). The actual segmentation process is an optimization problem in which a selected energy functional is minimized. The minimization can be realized by a gradient descent and represents the propagation of the contour from an initialization point $\Phi ({\mathbf x},t_0)$ to an optimum $\Phi ({\mathbf x},t_E).$ In our case, the gradient descent reads [31]

2 $$\begin{aligned} \frac{\partial {\Phi }}{\partial {t}} = H'(\Phi ) \Bigg [ ~\sum _{j=1}^M~ \underbrace{\mathrm {log}~\frac{p_{1j}(F_j)}{p_{2j}(F_j)}}_{\text { homogeneity term}} + \underbrace{\nu \cdot \mathrm {div}\frac{\nabla \Phi }{|\nabla \Phi |}}_{\text { curvature term}}~\Bigg ] \end{aligned}$$

where *H* is the Heaviside function ($H=0.5$ for $\Phi =0$, $H=0$ for $\Phi <0$ and $H=1$ for $\Phi >0$), $F({\mathbf x})$ the feature vector with *M* elements, and $p_{ij}$ the conditional probability density functions for the regions $\Omega _i$ ($i=\{1,2\}$). The first term in the equation allows to separate $\Omega _1$ and $\Omega _2$ based on the distribution of the feature values in those regions. The second term is the curvature term which controls the contour’s smoothness with $\nu =0.001|\Omega |^{0.7}$ being the weighting factor [33].

##### Adaption and contribution

Level set methods are powerful techniques that are beyond the scope of basic image processing [32]. Previous works often performed ROI selection by applying conventional image processing ideas, i.e. face detection and feature point tracking. Here, we exploit the benefits of level set segmentation to additionally consider novel findings regarding the cbPPG signal’s origin. Therefore, we defined homogeneity as essential selection criterion since the respective regions are less impacted by BCG effects [23, 24]. To achieve homogeneously illuminated ROIs, we included the image intensity values in the vector *F*. Furthermore, a texture measure $J({\mathbf x})$ was chosen to also avoid inhomogeneities in the skin’s surface topology which cause artifacts in case of motion [34]. We determined *J* by calculating the local standard deviations for each color channel in neighborhoods of $5\times 5$ pixels. The vector could then be formulated as $F:=(I_R, I_G, I_B, J_{RGB})$ where $J_{RGB}$ is the mean of the single texture images $J_R,$ $J_G,$ and $J_B.$ During the segmentation process, pixels are assigned to $\Omega _i$ based on the probability that the pixel’s intensity and texture values are similar enough to belong there. This probability was obtained using a Gaussian function [33]

3 $$\begin{aligned} p_{ij} = \frac{1}{\sqrt{2\pi \sigma _{ij}^2}}~\exp {\left( -\frac{(F_j-\mu _{ij})^2}{2\sigma _{ij}^2}\right) } \end{aligned}$$

in which $\mu _{ij}$ and $\sigma _{ij}$ are the mean and standard deviation of the values in $F_j(\mathbf {x})$ given that $\mathbf {x}\in \Omega _i$. One of the most crucial steps in our ROI selection algorithm is the initialization of the segmentation. In order to obtain homogeneous skin regions, we set the outcome of the skin classification $\Omega _{SKN}^{RGB}$ to $\Omega _1(t_0).$ The result $\Omega _{ROI}^{RGB}:=\Omega _1(t_E)$ represents our final ROI for the RGB image. Figure 2 depicts an example of a respective segmentation process.

#### Registration

A skin region, which appears homogeneous in the RGB image, might appear differently in the NIR image where LED spot lights were used. Therefore, we attempted to employ level set segmentation separately for the NIR image to find its most homogeneous skin regions. However, with $I_N$ being monochrome, the skin classifier was not applicable for initialization. The result from the RGB image could also not simply be assigned to the corresponding NIR image since the respective cameras had a different viewing angle in our setup (see Fig. 1). We decided to apply an intensity-based block-matching method to transfer $\Omega _{ROI}^{RGB}.$ Briefly, the green channel $I_G$ (less noisy than R and B channel) was divided into overlapping blocks of $5\times 5$ pixels at the ROI. For each block $\beta,$ the best matching block in $I_N$ was then determined within a search area $(d_x,d_y)$ around the block location of $I_G.$ The mean squared error (MSE) was chosen as the matching criterion [35]. Due to the different lighting conditions in the RGB and NIR video (see “Data and setup” section), we always mean adjusted the blocks that were compared. Therefore, the MSE reads

4 $$\begin{aligned} \mathrm {MSE} = \int _{\mathbf {x}\in \beta } [ (I_{G} - \mu _{G} \big ) - (I_{N}(x+d_x, y+d_y) - \mu _{N} ) ]^2~d\mathbf {x} \end{aligned}$$

where $\mu _{G}$ and $\mu _{N}$ are the block means. A priori knowledge about the cameras’ positioning allowed us to limit the search area to $d_x = [-60, 0]$ and $d_y = [0, 10]$ pixels. The outcome of the registration $\Omega _{REG}^{N}$ was set as the initialization state $\Omega _1(t_0)$ for the eventual segmentation process in which the feature vector read $F:=(I_N, J_N).$ The final ROI $\Omega _{ROI}^{N}$ was then defined by $\Omega _1(t_E).$

### Implementation and framework

The implementation of the presented method was realized in MATLAB R2016a. For the level set approach, we followed the suggestions by Osher and Fedkiw [32]. We shortly mention important aspects in that context but would like to refer the reader to their book for a detailed description. The partial differential Equation in (2) was solved numerically (forward Euler method) by an iterative procedure. The level set function $\Phi$ was initialized employing a signed distance function (see Fig. 2a) and reinitialized after each iteration step. The derivative of the Heaviside function $H'$ was replaced by a smooth delta function.

**Fig. 3:**
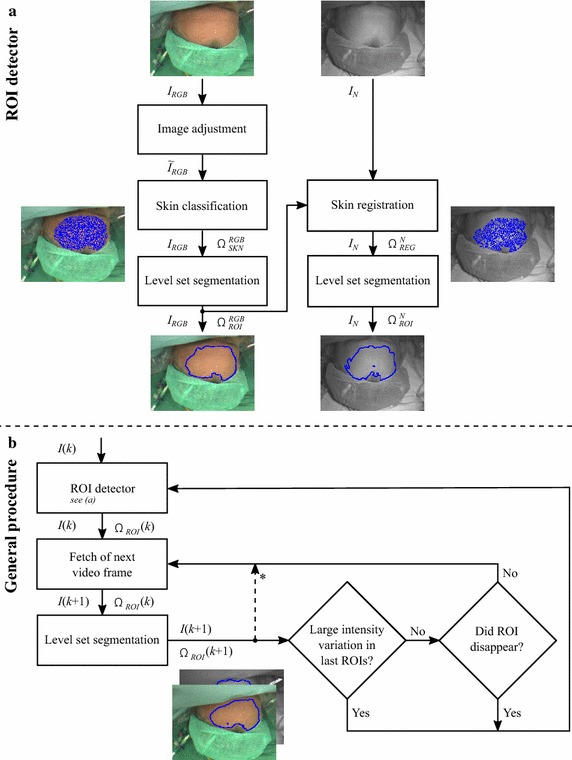
Program structure of the presented ROI detection and tracking algorithm.** a** ROI detector which (initially) detects the skin, finds the ROI and registers and adapts the result for the NIR image.** b** Simplified flowchart of the whole program (detection and tracking) which runs separately for the RGB and NIR video. For some transitions between the program blocks, the data types are given (*I*: image, $\tilde{I}$: adjusted image, $\Omega _{...}^{...}$: image region, ${k}$: frame number). * pause after ROI reselection

Figure 3b depicts the basic flow chart of our ROI selection method. An essential part is the *ROI detector* of which the program structure is shown in Fig. 3a. The detector’s principle components were already explained in the previous sections yielding two ROIs for a given image pair (e.g. for $k=1$). For the segmentation components, we used 300 (RGB image) and 100 iteration steps (NIR image) to obtain $\Omega _{ROI}^{RGB}$ and $\Omega _{ROI}^{N}$, respectively. These counts were determined empirically by selecting a broad variety of images and examining how many steps are at least necessary to reach a stabilized segmentation contour. The largest occurring step counts were rounded up and chosen for the whole data set. After detection, the ROIs were tracked separately in the RGB and NIR video streams. For that purpose, we also applied level set segmentation where the process for a frame was initialized by the ROI of the preceding frame: $\Omega _1(t_0,k):=\Omega _{ROI}(k-1)$ and $\Omega _{ROI}(k):=\Omega _1(t_E,k)$. Since possible changes between two consecutive frames are generally minor, only 50 iteration steps were necessary for convergence. In fact, when the contour remained nearly unchanged between two steps (regional size difference $\Delta |\Omega _1| < 50$ pixels), the segmentation was stopped early. The key idea behind the tracking approach was to rather track the intensity/ texture with their homogeneity inside the skin region than anatomical features. In this way, abrupt changes in the light intensity could be avoided within the ROI. However, certain artifacts, such as the temporary occlusion of the recording area by the staff, caused problems during tracking. Either the ROI was quickly assigned to non-suitable areas or disappeared completely because skin was no longer visible. The latter problem could be easily detected and was treated by executing the *ROI detector* repeatedly until skin regions were found again. To tackle the first problem, we always checked the mean intensity in the ROI for the last 10 s. If its standard deviation exceeded 50 units, our requirement of having stable ROI conditions was considered to be violated and the *ROI detector* was executed. As redetection might also lead to major intensity variations over time, after reselection, we paused the artifact monitoring for 10 s (see Fig. 3b).

### Signal processing

After image processing, the cbPPG signals were extracted by averaging the ROIs’ pixel values for each frame and color channel. As a result, we obtained four signals (R, G, B, NIR) for each patient throughout the recording. The signals were divided into consecutive 10 s segments amounting to an average of $192.2 \pm 43.5$ segments per subject and channel. Since ROIs could not always be selected (see previous section), the cbPPG signals occasionally held empty entries. Any segment that contained such entries was disregarded for the following steps. Each signal segment was removed from its linear trend and further filtered using an FIR highpass (order: 250, cutoff frequency: 0.5 Hz). Next, the signals were zero-padded to $2^{13}$ points, and the Fast Fourier transform was performed. Hence, we were able to determine a segment-wise HR by detecting the maximum peak in the related amplitude spectrum $|X(f)|$ within the range of 30 and 200 bpm. The same procedure was applied to calculate the reference HRs $f_{ref}$ out of corresponding 10 s segments in the PPG monitor signal. In order to assess the quality of the cbPPG signals, we computed the signal-to-noise ratio (SNR) by adapting a formula of de Haan and Jeanne [36]

5 $$\begin{aligned} \mathrm {SNR} = 10\cdot \mathrm {log}_{10}\left( \frac{\int _{f=30~\mathrm {bpm}}^{200~\mathrm {bpm}} \Pi \left( f \right) |X(f)|^2~df}{\int _{f=30~\mathrm {bpm}}^{200~\mathrm {bpm}} \left( 1-\Pi \left( f \right) \right) |X(f)|^2~df} \right) \end{aligned}$$

where $\Pi$ is defined as

6 $$\begin{aligned} \Pi (f) = {\left\{ \begin{array}{ll} 1 \quad&{} \mathrm {if} ~|f_{ref} - f| \le 5 ~\mathrm {bpm}\\ 1 \quad&{} \mathrm {if} ~|2 f_{ref} - f| \le 5 ~\mathrm {bpm}~.\\ 0 \quad&{} \mathrm {otherwise}\\ \end{array}\right. } \end{aligned}$$

The SNR considers the signal amplitudes around the true HR $f_{ref}$ and its first harmonic in a ± 5 bpm band as the wanted component and the remaining amplitudes between 30 and 200 bpm as the noise component.

### Evaluation and statistics

For each patient and color channel, signal processing provided between 103 and 368 HR and SNR values (dependent on recording time and artifacts) which were taken into account for evaluation. To analyze the two measures across all subjects, we built an individual HR detection rate (HDR) and a median SNR from those segment-related values. The HDR was determined as the relative number (in %) of HRs that deviated less than 5 bpm from the reference HRs. The segments which were excluded beforehand, due to missing ROIs, were treated as inputs where the HR was falsely detected.

Our overall goal was to show how well the proposed ROI selection method performs in an intraoperative environment. We did not focus on further transformation techniques (e.g. source separation) to achieve the best possible HDR. Therefore, we assessed the results separately for each color channel. However, we regarded the NIR channel to be of special interest since a dedicated illumination setup was applied. For this reason, we tested whether the combination of the channel with the best performing channel (here green) yields a significantly better HDR outcome than only using the green channel. We also evaluated the combinations G&B and G&R for reference purposes. The HDR values of a combination resulted from the assumption that for a segment, always the correct HR (if available) can be selected between the two considered channels. The significance of the improvements was analyzed by employing a Wilcoxon sign rank test (one-tailed) as follows: G to G&B, G to G&R, and G to G&NIR.

## Results

### ROI selection

**Fig. 4:**
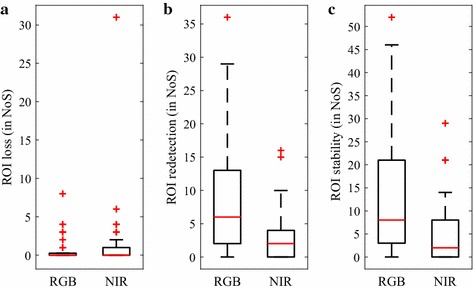
Reliability metrics of the ROI selection process.** a** Number of segments (NoS) per patient in which single ROIs were absent.** b** NoS in which the *ROI detector* had to be re-executed.** c** NoS in which the standard deviation of the mean ROI intensity exceeded 50 (see “Implementation and framework” section). Each boxplot depicts 41 patient-related values

For all 41 patients, appropriate ROIs were automatically detected and tracked in both, the RGB and NIR video. As mentioned before, in some rare cases, the ROI was not determinable. For the RGB and the NIR videos, the average numbers of segments, which were affected by the absence of single ROIs, were generally low reaching a maximum of 8 and 31, respectively (see Fig. 4a). A further quality attribute of our method is how often the *ROI detector* had to be re-executed. Regarding the median value, in only 6 segments of the RGB videos and 2 segments of the NIR videos, the ROI was redetected over the duration of the recording (see Fig. 4b). In the “Implementation and framework” section, it was described that the ROI stability was considered compromised if the standard deviation of the mean ROI intensity exceeded 50. Figure 4c visualizes the respective segment counts proving an overall low ROI fluctuation.

**Fig. 5:**
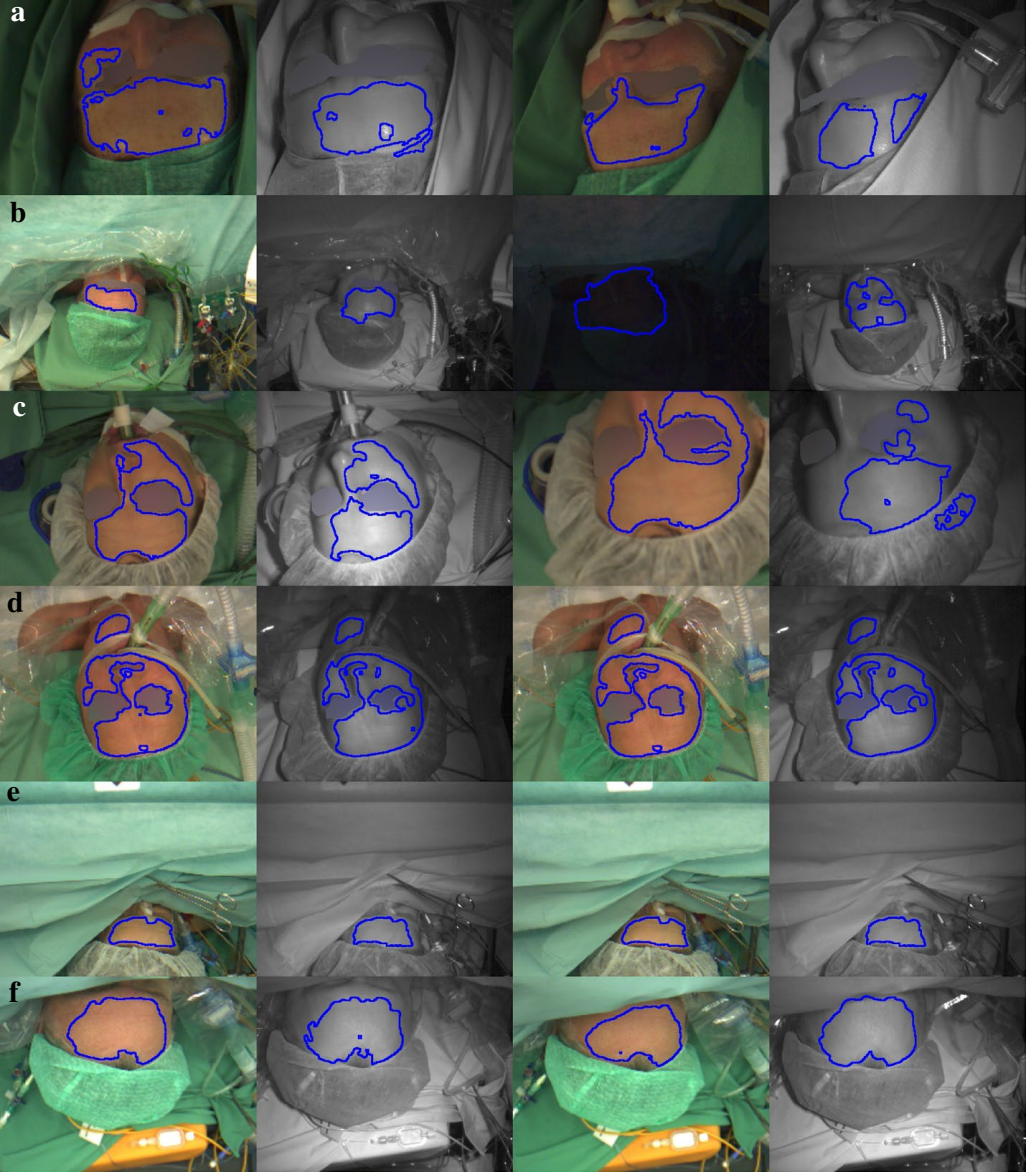
Selected ROIs for six different patients. The first two columns show the ROIs (only contour) for the RGB and NIR image at the beginning of the recording, the last two columns at a later point. If there was minor or no movement, the results in column 1 and 2 are similar to those in 3 and 4. Please note that in case the patient was identifiable, the eye section in the depicted images was blurred

Figure 5 shows the selected ROIs of six patients at different states in the videos. The examples represent the strength of our approach being robust against illumination changes, limitations in the face’s visibility, and against variations in scale and rotation. All ROIs contain homogeneously illuminated skin regions which demonstrate our method to reject relatively darker regions and regions that were not orthogonally aligned towards the camera (see Fig. 5a, c). Moreover, an ROI can consist of several unconnected regions and may have holes serving the purpose of homogeneity (see Fig. 5a, c, d). In Additional file 1 of this article, a video is linked which visualizes the described performance for an example. The advantage of using a separate segmentation step for the NIR image in the *ROI detector* is comprehensible when looking at Fig. 5a–c. The lighting situation in the NIR video was considerably different from the one in the RGB video. Therefore, a simple ROI registration based on the head’s pose would not have been sufficient since homogeneous areas were required.

We also tested the real-time capability of our method. Solely the *ROI detector* needed longer processing times of about 10 s (MATLAB, i5-4590 @ 3.3 GHz on a single core). The tracking could be performed in real-time ($<10$ ms). In this study, we did not focus on creating an online method. Nevertheless, prospective works could speed up the algorithm to that end by implementing it in C++ and taking advantage of parallel computing.

### HR detection and SNR

**Fig. 6:**
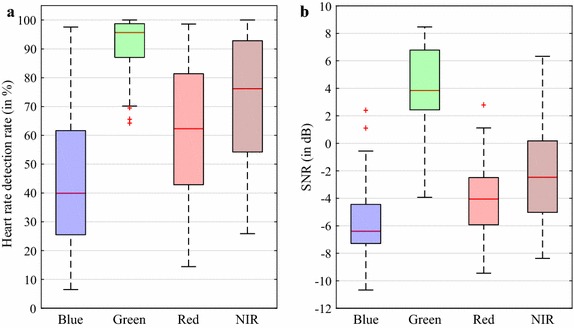
Results of cbPPG measures when using the proposed method.** a** Heart rate detection rate for the red, green, blue and near-infrared channel.** b** Signal-to-noise ratio (SNR)

Figure 6a depicts the results of the HDR for the four color channels. Across all patients, the green channel provided the best outcome when applying our method (median of 95.6%). The NIR channel yielded a moderate detection rate (median of 76.2%) while the red and the blue channel are rather poor candidates to correctly detect the HR (median of 62.3 and 39.9%). The variation among the patients was the lowest for the green channel leaving only a small number of subjects with lower HDR values. Figure 6b shows the results of the SNR. As can be derived from the plot, the HDR is related to the quality of the cbPPG signals where the green channel also generates the best outcome (median of 3.9 dB) followed by the NIR, red, and blue channel in order of performance (median of − 2.5, − 4.1, and − 6.4 dB). However, in contrast to the HDR, the variation among the individual SNR values proved to be higher for the better performing channels.

**Fig. 7:**
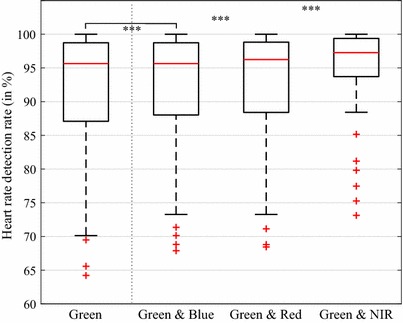
Heart rate detection rate for the green channel in comparison to channel combinations. The combinations are determined assuming that always the correct heart rate (if available) can be selected between the two channels. Each boxplot depicts 41 patient-related values. The outcome of the statistical tests is shown above the boxes (***$p<0.001$)

In the previous section, we explained our attempt to explore what contribution the blue, the red, and particularly the NIR channel might make within our method. The results reveal all considered channel combinations to yield significantly higher HR detection rates than the green channel alone (see Fig. 7). As presumed, the combination with the NIR channel involved the largest improvement in the median HDR (95.6 versus 97.3%). Furthermore, except of a few outliers, all patients showed rates above 88% in the G&NIR group while in the other groups, a relatively large number of subjects lay under 80%. In 29 of the 41 patients, the NIR channel was able to provide at least once and up to 22 times a correct HR (average of 4.6 segments) when all the other channels failed.

## Discussion

### ROI selection

Skin classifiers are an easy way to locate potential ROIs. For classification, most works in cbPPG applied absolute thresholds in the components of various color spaces, most often of the YCbCr space [17–21, 37]. We tested this classifier in our framework. The given thresholds led to a general overrepresentation of the skin areas, and we found it hard to adjust to changing conditions on a large scale of data. The used Bayesian classifier was trained with pictures that comprised numerous skin tones captured in different environment and illumination situations. Although it was barely employed for cbPPG so far [12, 38], we found it to be robust and its outcome to be well-controllable ($\theta$ adjustment). We tested that higher $\theta$ values (low false positive but also low true positive rate) lead to better ROIs since the classifier is only used to initialize the segmentation method which is able to compensate an underrepresentation of the skin (see Fig. 2). Level set segmentation is an iterative process where the evolving contour has to reach a stable state. For the RGB images, stabilization was usually not an issue because the information of three color channels allowed a clearer separation. For the NIR images, more problems occurred. In rare cases, the contour increased or decreased uncontrollably. Additional knowledge about potential skin areas, e.g. by using $p({\mathbf c} | skin)$ in *F*, could solve those problems. However, it would require a reliable mapping of the RGB data on the NIR images.

Homogeneity is an important criterion in ROI selection. Rodríguez and Castro [25] applied a simple intensity threshold to exclude darker areas like the eyebrows. Yang et al. [9] built a roughness measure in sub-ROIs which was employed to select the smoothest regions. Bousefsaf et al. [26] used the lightness component of the CIE L*u*v space to create five regional clusters of which the best were eventually combined. Yet, none of these methods allowed a continuous (time and space) pixel-wise selection as it could be accomplished by level set segmentation.

**Fig. 8:**
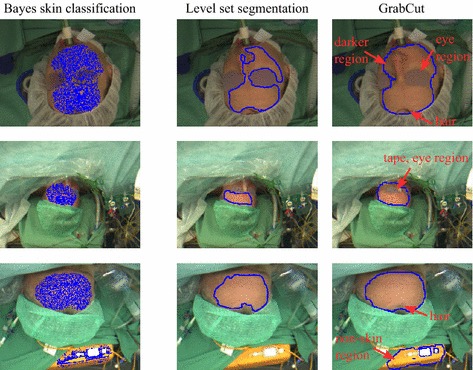
Comparison of the proposed method to a GrabCut-based approach. Three examples (RGB video) are depicted in the state of the initial ROI detection. The first column shows the result of the skin classifier. Similar as in our method, it was used as initialization for *GrabCut* although morphological closing was performed beforehand (see [39]). The last two columns show the final ROIs (only contour) in which the red arrows highlight the lack of performance of *GrabCut*. Please note that in case the patient was identifiable, the eye section in the depicted images was blurred. Due to eyebrows, eyelashes, and shadowing effects, the region around the eyes usually appears darker than the surrounding area

Besides homogeneity, another advantage of our approach is that it neither depends on anatomical features nor on the manifestation of the cardiac pulse. There are only a few works which fall into this category. Wang et al. [20, 21] exclusively applied a skin classifier (see above) for ROI detection. Potential insufficiencies in the outcome, however, were disregarded as the group focused on signal processing. Similar to our procedure, Stricker et al. [39] employed skin classification in combination with a segmentation method, namely *GrabCut* [40]. Due to the resemblance, we decided to test the method for a number of images in our setting (see Fig. 8). We followed the description of the authors in which the result of the skin detector was first morphologically closed and then used for initialization in *GrabCut*. In comparison to our method, the *GrabCut*-based approach showed a systematic lack of performance as high-contrast non-skin and more heterogeneous skin areas were selected.

### HR detection and SNR

The SNR assesses the cbPPG signals’ quality based on the HR. The response characteristic of the different wavelengths coincides with the outcome of prior investigations regarding the quality of photoplethysmograms [41]. As a higher quality involves a stronger manifestation of the cardiac pulse, the chances of correctly detecting the HR also increase (see similarities in Fig. 6a, b). Nevertheless, the SNR measure has limitations since the stated relation not always holds and a high HDR can be associated with a low SNR (see high variance in SNR plots). In general, the proposed method is able to select ROIs which provide cbPPG signals (green channel) that largely show a distinct pulsation and are scarcely degraded by artifacts. To a small degree, false HR detections are attributed to cases where no ROIs were found. The majority of false detections can be explained by situations when the *ROI detector* was re-executed. Our tracking idea was to retain the regions’ homogeneity and avoid abrupt light changes. However, the reselection of the ROI does not consider prior intensity values and may lead to an edge in the cbPPG signal hindering a valid HR extraction.

**Fig. 9:**
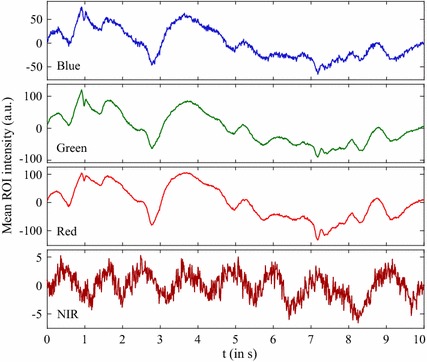
Signal examples where artifacts occurred. Related signal segments for the R, G, B and NIR channel where the HR was detected correctly solely in the NIR signal. The ROIs were well-defined in both videos. Light variations in the ambient light caused artifacts to occur in the RGB video while the NIR video remained unaffected (cardiac pulse is visible). Please note that the strength of the pulsatile component usually does not exceed $\pm 15$ units for the set color depth

The NIR channel played a special role in our investigation since a separate camera and light source was used. Estepp et al. [42] already demonstrated that a multi-camera setting can enhance the HDR. In our setting, the dedicated NIR illumination yielded stable conditions in moments where the ambient light was low or strongly altered (see Figs. 5b and 9). Therefore, the NIR channel also made the highest contribution to maximizing the HDR (see Fig. 7). However, the problem of accurately mapping the ROI from the RGB to the NIR image remains. The application of cameras with a native alignment between the RGB and NIR channels (e.g. [43]) resolves this drawback.

Moço et al. [23, 24] revealed how homogeneously illuminated regions provide purer cbPPG signals that are less corrupted by BCG artifacts. Our method is able to select such regions. Furthermore, it is an alternative to the group’s methods, which also dealt with those artifacts but had to be calibrated beforehand.

We would like to emphasize again that we aimed at demonstrating the high performance of our ROI selection approach and not necessarily at reaching a maximum HDR. However, if certain applications require a reliable HR detector, appropriate signal processing steps can be subsequently executed. We tested that solely a simple principal component analysis on the R, G, B channel signals leads to detection rates over 99%.

### Intraoperative setting

To the best of our knowledge, we are the first to apply cbPPG during surgery with the patients being under general anesthesia. Rubīns et al. [27, 28] investigated the effect of vasodilation in the course of regional anesthesia using cbPPG, once in the NIR light range and once in the green range. Both times, they considered the inner region of a fixed hand (no movement) and built amplitude maps, which did not demand a prior ROI selection but presumed the presence of cardiac pulsations in signals from spatial subregions.

## Conclusions

In this paper, we presented a fully automated ROI selection method for cbPPG. It overcomes the drawbacks of past approaches and, therefore, allowed us to employ cbPPG in vascular diseased patients in an intraoperative environment. The method neither relies on the visibility of anatomical features nor on the manifestation of the cardiac pulsation. Homogeneity in intensity and texture are the determining criteria for choosing and tracking ROIs. As a result, distinct and mostly undistorted photoplethysmograms could be obtained. Our method is easily transferable to other applications where other body sites are involved. Moreover, it can be run for multi-camera systems as long as one RGB camera is part of the setting. Eventually, the method enables prospective studies to focus on the benefit of using cbPPG during surgery. The spatial assessment of the cutaneous microcirculation might help the anesthetists to better react to cardiovascular events and adjust the respective medication.

## Additional file

**Additional file 1.** A video showing the application of the proposed method. The video shows a moving face for which the proposed method was applied in order to select an ROI. For comparison purposes, the Viola-Jones face detector combined with the KLT feature tracker was employed [1, 2]. In contrast to this standard approach, our method only chooses homogeneously illuminated skin regions that are most suitable for cbPPG.
1. Viola, P., Jones, M.: Rapid object detection using a boosted cascade of simple features. Proceedings of the 2001 IEEE Computer Society Conference on Computer Vision and Pattern Recognition, vol. 1, pp. I-511–I-518 (2001).
2. Tomasi, C., Kanade, T.: Detection and Tracking of Point Features. Technical Report MU-CS-91-132, Carnegie Mellon University (1991).

## Abbreviations

cbPPG: camera-based photoplethysmography
ROI: region of interest
RGB: red, green, blue
NIR: near-infrared
BCG: ballistocardiographic
HR: heart rate
LED: light emitting diode
PC: personal computer
MSE: mean squared error
FIR: finite impulse response
SNR: signal-to-noise ratio
HRD: heart rate detection rate
NoS: number of segments

## Footnotes

1 By applying MATLAB’s function imadjust(), the intensity values of each channel were mapped so that the whole range (0–4095) was used.
